# Welcome to the New Journal *Methods and Protocols*

**DOI:** 10.3390/mps1010001

**Published:** 2017-06-27

**Authors:** Fernando Albericio

**Affiliations:** 1 School of Chemistry, University of KwaZulu-Natal, Durban 4001, South Africa; albericio@ukzn.ac.za; 2 Department of Organic Chemistry, University of Barcelona, 08028-Barcelona, Spain

I am pleased to introduce *Methods and Protocols*, a new multidisciplinary, peer-reviewed, open access journal devoted to providing an open forum to report on new procedural approaches and cutting-edge methodological developments. We aim to disseminate the latest experimental and technical developments to provide authors with new tools that can be readily adopted in their research.

The scope of *Methods and Protocols* is broad, covering experimental approaches to tackle questions in various scientific fields, including but not limited to Life Sciences, Chemistry, Biomedical Sciences and Engineering. Scientists are encouraged to prepare videoarticles to be published alongside textual articles in order to increase the reproducibility and visibility of newly developed protocols/methods. I expect *Methods and Protocols* to become a respected venue for the peer-reviewed, rapid and affordable publication of papers about disruptive experimental approaches and developments.

To kick off *Methods and Protocols*, we will launch an ambitious series of Special Issues covering selected topics of high current relevance, including protocols, benchmarks (methods) and short reviews. Example topics include advances in genome engineering, soil microbiome proteomics and metabolomics, bacteriophage research and applications, and gene editing in non-standard model organisms. We welcome suggestions for topics for Special Issues, which will be edited by proficient researchers in their respective fields in order to set a high standard for the journal and achieve high visibility for its publications.

On behalf of the Editorial Board and the Editorial Office, I welcome all authors, reviewers and external editors, and thank them for joining us in this exciting project and for their contributions to develop *Methods and Protocols*. I anticipate that this journal will foster fruitful crosstalk and cross-fertilization between the various disciplines covered by the journal.

## Supplementary Materials

Supplementary File 1

